# Factors Associated with Self-reported COVID-19 Infection and Hospitalization among Patients Seeking Care at a Comprehensive Cancer Center

**DOI:** 10.1007/s40615-023-01855-4

**Published:** 2023-11-02

**Authors:** Rossybelle P. Amorrortu, Yayi Zhao, Robert J. Keenan, Scott M. Gilbert, Dana E. Rollison

**Affiliations:** 1 Department of Cancer Epidemiology, Moffitt Cancer Center, 12902 Magnolia Drive, CSB 8th 8108, Tampa, FL 33612 USA; 2 Department of Thoracic Oncology, Moffitt Cancer Center, Tampa, FL USA; 3 Department of Health Outcomes and Behavior, Moffitt Cancer Center, Tampa, FL USA; 4 Department of Genitourinary Oncology, Moffitt Cancer Center, Tampa, FL USA

**Keywords:** COVID-19, Self-report, Infections, Hospitalization, Risk Factors, Epidemiology

## Abstract

**Background:**

COVID-19 infection severity differs by race and ethnicity, but its long-term effect on cancer-related outcomes is unknown. Therefore, information on COVID-19 history is critical to ascertain among new cancer patients in order to advance research on its impact on cancer outcomes and potentially related health disparities.

**Methods:**

A cross-sectional study was conducted among 16,025 new patients seeking care at Moffitt Cancer Center (MCC) between 2021 and 2022. Patient self-reported histories of COVID-19 infection and other pre-existing health conditions were obtained from electronic questionnaires administered to all new MCC patients. Associations between demographics and COVID-19 infection and hospitalization were examined.

**Results:**

A total of 1,971 patients (12.3%) reported ever having COVID-19. Self-reported COVID-19 history was significantly more prevalent in Hispanic vs. non-Hispanic patients (OR = 1.24, 1.05–1.45) and less prevalent in Asian versus White patients (OR = 0.49, 95% 0.33–0.70). Among patients who ever had COVID-19, 10.6% reported a COVID-19-related hospitalization. Males had higher odds of a COVID-19 related hospitalization than females (OR = 1.50, 95% CI = 1.09–2.05), as did Black/African American patients (OR = 2.11, 95% CI = 1.18–3.60) and patients of races other than Black/African American and Asian (OR = 2.61, 95% CI = 1.43–4.54) compared to White patients. Hispanic patients also experienced higher odds of hospitalization (OR = 2.06, 95% CI-1.29- 3.23) compared with non-Hispanic patients of all races in a sensitivity analysis that combined race/ethnicity. Pre-existing lung and breathing problems were associated with higher odds of being hospitalized with COVID-19 (OR = 2.38, 95% CI = 1.61–3.48), but these and other health conditions did not explain the observed associations between race and COVID-19 hospitalization.

**Conclusions:**

Higher rates of COVID-19 hospitalization were observed among patients identifying as Black/African American or Hispanic independent of pre-existing health conditions. Future studies evaluating long-term effects of COVID-19 should carefully examine potential racial/ethnic disparities in cancer outcomes.

**Supplementary Information:**

The online version contains supplementary material available at 10.1007/s40615-023-01855-4.

## Background

A novel coronavirus type was identified in 2019 and named the severe acute respiratory coronavirus-2 (SARS CoV-2) [1–3]. The SARS CoV-2 virus rapidly spread globally resulting in the “COVID-19 pandemic” [4] causing major morbidity and mortality with over 760 million cases and 6.9 million deaths reported world-wide as of April 23, 2023 [5]. In the United States, the number of daily cases (7-day average) ranged from 177,520 in January in 2021 to over 800,000 cases in June of 2022 [6]. Deaths from COVID-19 disproportionately impacted Black/African American and Hispanic individuals [7–9]. Other factors associated with COVID-19 severity include older age, male sex, and pre-existing health conditions such as hypertension, cardiovascular diseases, diabetes, chronic kidney disease, respiratory or metabolic diseases [10–13].

Overall, the COVID-19 pandemic led to major disruptions in clinical care, including cancer care [14], such as interference with hospital operations, postponement of doctors’ visits and cancer screenings, treatment delays or treatment discontinuation, and cancellation of elective surgeries [15–18]. Previous studies have reported worse overall outcomes in patients with cancer including more severe COVID-19 symptoms, COVID-19 related complications, and increased COVID-19 related mortality [19–22]. However, much is still unknown about the longer-term effects of COVID-19 on cancer treatment response, recurrence, and overall survival [13–16]. Importantly, some recent laboratory studies suggest that COVID-19 infection may generate autophagy in cancer cells leading to poor cancer outcomes such as progression or recurrence [23, 24]. Therefore, assessment of baseline information on prior COVID-19 infection among new patients within a cancer center is critical for future research on the potential impact of prior COVID-19 infection on subsequent cancer treatment response and outcomes, as well as associated health disparities.

Moffitt Cancer Center (“Moffitt”) incorporated questions on history and severity of COVID-19 within the Electronic Patient Questionnaire (EPQ), a clinical intake form presented to new patients. The purpose of this study was to examine the prevalence of self-reported COVID-19 infection and hospitalization and their associations with age, race, ethnicity, gender, and pre-existing health conditions among patients seeking care at a comprehensive cancer center. Results from this study will inform future research on COVID-19, cancer treatment outcomes and associated health disparities.

## Methods

### Study Methods and Population

A cross-sectional analysis of de-identified data was conducted to examine the associations between demographic characteristics, history of cancer, pre-existing health conditions and self-reported COVID-19 infection and hospitalization among 16,025 adult patients new to Moffitt who completed the EPQ between January 2021-February 2022. The Moffitt Scientific Review Committee reviewed the study protocol (MCC#22,268) and determined that the study met the definition of non-human subject’s research and did not require IRB approval.

### Data Collection

Detailed data collection methods are described elsewhere [25]. Briefly, the EPQ is a new patient intake form made available for all new patients to complete within the patient portal once their first visit is scheduled. Patients who do not complete the EPQ prior to their first visit are asked to complete the questionnaire on a tablet in the clinic. EPQ responses are incorporated into the electronic medical record and are available for research via the Moffitt data warehouse. The EPQ includes questions on demographics, current cancer diagnosis (if applicable), personal and family medical history, cancer risk factors, current symptoms, and quality of life. Pertinent to the current analysis, patients are asked about pre-existing non-cancer health conditions using the following question: “Other than the cancer diagnoses that you may have already reported, have you ever been diagnosed with any other medical problem, disease, or condition?”. Patients are then presented with a list of conditions to select from, with the ability to select multiple conditions or to indicate that they did not have any of the listed conditions (Online Resource 1).

An advantage of the EPQ is that it can be easily modified to collect timely data at scale. For example, in January 2021, questions were added to the EPQ to ascertain patients’ history of COVID-19 infection (prior to the widespread availability of COVD-19 self-testing). Specifically, patients are asked if they “ever had COVID-19” and respondents who answered “yes” were subsequently asked “Was your COVID-19 confirmed with a diagnostic test?” and “Were you hospitalized for your COVID-19?”.

EPQ responses pertaining to personal medical history, including COVID-19 infection, were obtained from Moffitt’s data warehouse, as were demographic data derived from the electronic health record (Cerner), including age at questionnaire completion, sex, race, and ethnicity. The race/ethnicity variables were documented as two separate variables within Cerner and defined in accordance with the Office of Management and Budget standards and the North American Association for Centralized Cancer Registries.

### Statistical Analysis

Descriptive statistics were used to summarize patient characteristics, including age at questionnaire completion, sex, race, ethnicity, prevalent or (history of) cancer, history of organ transplantation, and pre-existing health conditions. Prevalence of cancer history and pre-existing health conditions were reported separately for groups defined by COVID-19 infection (ever/never) and among those who reported ever having COVID-19 infection, by hospitalization for COVID-19 infection (yes/no).

As a first step, exploratory multi-variable logistic regression models were used to evaluate whether the most common pre-existing health conditions were associated with the odds of a previous COVID-19 infection, as well as with the odds of hospitalization for COVID-19 among those infected. In the first exploratory model, a backward elimination process was conducted on the 10 most prevalent pre-existing health conditions while treating self-reported COVID-19 infection as the outcome variable to remove pre-existing health conditions that were not significantly associated with COVID-19 infection (p > 0.05). The second exploratory model was constructed using the same list of pre-existing health conditions as predictors while treating COVID-related hospitalization as the outcome. Any pre-existing health conditions that were retained in either exploratory model were carried forward and served as covariates in the construction of the final multi-variable models.

To construct a final model for COVID-19 infection, we started with a full list of covariates that were taken into consideration, including demographic factors, history of cancer, and the previously identified pre-existing health condition. Subsequently, a backward elimination process was conducted on the full model to remove predictors that were not significantly associated with COVID-19 infection after adjusting for other factors (p > 0.05). A similar process was conducted to derive the final model for hospitalization due to COVID-19 infection.

To further tease apart the associations with race and ethnicity, we conducted a sensitivity analysis by modeling race and ethnicity as a single variable including four levels: non-Hispanic White (reference), non-Hispanic Asian, non-Hispanic Black/African American, and Hispanic. All analysis were conducted using R version 4.21 [26].

## Results

A total of 16,025 new patients answered the COVID-19 related EPQ questions between January 2021-February 2022, of whom 50.2% were 65 years or older, 55.5% were female, 82.3% were White, and 84.1% were non-Hispanic (Table 1). Regarding COVID-19 positivity, a total of 12.3% of patients reporting ever having COVID-19 infection, of whom 88.9% reported their COVID-19 infection was confirmed by a diagnostic test (Table 1). Among the 1,971 patients who self-reported ever having COVID-19, 10.6% of patients reported a COVID-19-related hospitalization. Of note, when examining these trends over time the COVID-19 infection rate increased from 8.6% in the first half of 2021 to 17.5% in early 2022 while the hospitalization rates decreased from 10.6 to 3.4%.

**Table 1:** Patient demographic characteristics and self-reported history of cancer and pre-existing health conditions among 16,025 patients who completed the EPQ between Jan 2021-Feb 2022

Patient Characteristics	n (%)
Age at survey (years)	
18–25	274 (1.7)
26–35	727 (4.5)
36–45	1,416 (8.8)
46–55	2,215 (13.8)
56–64	3,344 (20.9)
65–74	4,702 (29.3)
75 +	3,347 (20.9)
Sex	
Female	8,896 (55.5)
Male	7,124 (44.5)
Missing	5 (0.0)
Race	
White	13,194 (82.3)
Asian	412 (2.6)
Black/African American	989 (6.2)
American Indian or Alaska Native	43 (0.3)
Native Hawaiian or Other Pacific Islander	29 (0.2)
Other race	443 (2.8)
More than 1 race	120 (0.7)
Missing	795 (5.0)
Ethnicity	
Non-Hispanic	13,474 (84.1)
Hispanic	1,403 (8.8)
Missing	1,148 (7.2)
Self-reported history of cancer	
No	4,991 (31.1)
Yes	11,010 (68.7)
Missing	24 (0.1)
Self-reported history of organ transplantation	
No	15,986 (99.8)
Yes	15 (0.1)
Missing	24 (0.1)
Self-reported pre-existing health conditions	
Any of the below health conditions	13,623 (85.1)
Depression, anxiety, or other mental health problems	3,220 (20.1)
Allergies, sinuses, or hay fever	5,259 (32.9)
Arthritis, autoimmune diseases, or joint problems	4,760 (29.8)
Bleeding, clotting, or other blood problems	1,331 (8.3)
Breast or nipple problems	673 (4.2)
Diabetes, thyroid, or gland problems	3,980 (24.9)
Gyn (gynecological) problems including problems with uterus, ovaries, vagina, vulva, and cervix (female only)	2,067 (23.5)
Heart problems or high blood pressure	6,567 (41.0)
Kidneys, bladder, adrenal gland, or urinary tract problems	2,394 (15.0)
Lung or other breathing problems	2,065 (12.9)
Brain and neurological system problems such as stroke, headaches, or seizures	1,458 (9.1)
Prostate problems (male only)	1,639 (22.9)
Skin or mole related problems	2,743 (17.1)
Digestive tract problems including stomach, colon, bowels, pancreas, liver, and gallbladder	3,599 (22.5)
Penis, testis, or sperm problems (male only)	267 (3.7)
Other health problems	1,422 (8.9)
History of COVID-19 infection	
Never had COVID-19	14,054 (87.7)
Ever had COVID-19	1,971 (12.3)
COVID-19 infection confirmed by a diagnostic test^a^	
No	218 (11.1)
Yes	1,753 (88.9)
COVID-19 related hospitalization^a^	
No	1,762 (89.4)
Yes	209 (10.6)

^a^Among participants who reported to ever having COVID-19

At the time of EPQ completion, 68.7% of the patients reported having or having had cancer, with the remainder not yet having received a cancer diagnosis at the time of the intake questionnaire completion. A prevalent (or history of) cancer was slightly more common among patients who never reported having a COVID-19 infection (69.4% among those who never had COVID-19 vs. 64.5% among those who ever had COVID), with no major differences observed by cancer type (Online Resource 2). In terms of pre-existing health conditions, 85.1% of patients reported having at least one condition (Table 1). The most frequently reported conditions among this patient population were heart problems or high blood pressure (41.0%), allergies, sinuses, or hay fever (32.9%), arthritis, autoimmune diseases, or joint problems (29.8%), and diabetes, thyroid, or gland problems (24.8%). A history of organ transplantation was reported in 0.1% of the patient population. In general, a higher prevalence of pre-existing health conditions was observed among patients who never had a COVID-19 infection (85.7%) as compared with those who had the infection (81.5%) (Online Resource 3). However, among patients who had a COVID-19 infection, those who were hospitalized reported a higher prevalence of any pre-existing health condition as compared with those who never had a COVID-19 related hospitalization (Online Resource 3).

Associations between patient characteristics and COVID-19 infection are shown in Table 2. In the unadjusted analysis, history of COVID-19 was inversely associated age, with 22.3% of patients ages 18–25 reporting a history of COVID-19, compared to 7.2% of those ages 75 and older (odds ratio [OR] = 0.27, 95% confidence interval (CI) = 0.19–0.37). Females (13.1%) did not differ significantly from males (11.3%) with respect to history of COVID-19. Differences in prevalence of COVID-19 history were observed by race, ranging from 6.9% in American Indian/Alaskan Native patients to 19.4% in patients who reported an other race, and by ethnicity, with a higher prevalence of COVID-19 observed among Hispanic patients (17.8%) compared to non-Hispanic patients (12.0%). Patients with a history of cancer and those with certain pre-existing health conditions had lower rates of COVID-19 infection including heart problems or high blood pressure, diabetes, thyroid or gland problems, and lung or other breathing problems (Table 2). However, in the multivariable analysis, the only variables retained were age, race, and ethnicity, with all pre-existing health conditions dropped from the model. After adjustment for age and ethnicity, Asian patients had lower rates of COVID-19 infection than White patients (7.5% vs. 12.3%, OR = 0.49, 95% CI = 0.33–0.70). Black/African American patients also had lower odds of COVID-19 infection than White patients (OR = 0.85, 95% CI = 0.70–1.04), although this finding did not reach a statistical significance. After adjustment for age and race, Hispanic patients reported a history of COVID-19 infection more often than non-Hispanic patients (17.8% vs. 12.0%, OR = 1.24, 95% CI = 1.05–1.45). Similar patterns were observed for COVID-19 infections reportedly confirmed by a diagnostic test (Table 2).

**Table 2:** Self-reported history of COVID-19 infection by patient characteristics among new Moffitt Cancer Center patients in 2021–2022

Patient Characteristics^a^	Self-reported COVID-19 Infection								
	Never had COVID-19	Ever had COVID-19		Unadjusted odds ratios	Multivariable odds ratios	COVID-19 confirmed by diagnostic test		Unadjusted odds ratios	Multivariable odds ratios
	n	n	%^b^	(95% CI)	(95% CI)	n	%^c^	(95% CI)	(95% CI)
Age at survey									
18–25	213	61	22.3	1.00 (ref.)	1.00 (ref.)	55	20.5	1.00 (ref.)	1.00 (ref.)
26–35	570	157	21.6	0.96 (0.69–1.35)	0.95 (0.67–1.35)	141	19.8	0.96 (0.68–1.37)	0.96 (0.67–1.39)
36–45	1,160	256	18.1	0.77 (0.57–1.06)	0.77 (0.56–1.08)	225	16.2	0.75 (0.54–1.05)	0.76 (0.55–1.09)
46–55	1,822	393	17.7	0.75 (0.56–1.03)	0.75 (0.55–1.03)	352	16.2	0.75 (0.55–1.04)	0.75 (0.54–1.06)
56–64	2,908	436	13.0	0.52 (0.39–0.71)	0.53 (0.39–0.74)	377	11.5	0.50 (0.37–0.69)	0.52 (0.38–0.74)
65–74	4,274	428	9.1	0.35 (0.26–0.48)	0.34 (0.25–0.47)	387	8.3	0.35 (0.26–0.48)	0.35 (0.26–0.50)
75 +	3,107	240	7.2	0.27 (0.20–0.37)	0.27 (0.19–0.37)	216	6.5	0.27 (0.20–0.38)	0.27 (0.20–0.39)
Sex									
Female	7,728	1,168	13.1	1.00 (ref.)	1.00 (ref.)	1,047	11.9	1.00 (ref.)	
Male	6,322	802	11.3	0.84 (0.76–0.92)	DROPPED	705	10.0	0.82 (0.74–0.91)	DROPPED
Missing	4	1	20.0			1	20.0		
Race									
White	11,565	1,629	12.3	1.00 (ref.)	1.00 (ref.)	1,437	11.1	1.00 (ref.)	1.00 (ref.)
Asian	381	31	7.5	0.58 (0.39–0.82)	0.49 (0.33–0.70)	31	7.5	0.65 (0.44–0.93)	0.56 (0.37–0.80)
Black/African American	857	132	13.3	1.09 (0.90–1.32)	0.85 (0.70–1.04)	121	12.4	1.14 (0.93–1.38)	0.89 (0.72–1.09)
American Indian or Alaska Native	36	7	16.3	1.45 (1.17–1.79)	1.01 (0.79–1.28)	7	16.3	1.56 (1.25–1.93)	1.07 (0.84–1.37)
Native Hawaiian or Other Pacific Islander	27	2	6.9			2	6.9		
Other race	357	86	19.4			82	18.7		
More than 1 race	107	13	10.8			11	9.3		
Missing	724	71	8.9			62	7.9		
Ethnicity									
Non-Hispanic	11,852	1,622	12.0	1.00 (ref.)	1.00 (ref.)	1,430	10.8	1.00 (ref.)	1.00 (ref.)
Hispanic	1,153	250	17.8	1.58 (1.37–1.83)	1.24 (1.05–1.45)	236	17.0	1.70 (1.46–1.97)	1.31 (1.11–1.55)
Missing	1,049	99	8.6			87	7.7		
Self-reported cancer									
No	4,292	699	14.0	1.00 (ref.)	1.00 (ref.)	628	12.8	1.00 (ref.)	
Yes	9,740	1,270	11.5	0.80 (0.73–0.88)	DROPPED	1,123	10.3	0.79 (0.71–0.87)	DROPPED
Missing	22	2	8.3			2	8.3		
Self-reported heart problems or high blood pressure^d^									
No	8,138	1,295	13.7	1.00 (ref.)	1.00 (ref.)	1,143	12.3	1.00 (ref.)	
Yes	5,893	674	10.3	0.72 (0.65–0.79)	DROPPED	608	9.4	0.73 (0.66–0.81)	DROPPED
Missing	23	2	8.0			2	8.0		
Self-reported diabetes, thyroid, or gland problems^d^									
No	10,480	1,540	12.8	1.00 (ref.)	1.00 (ref.)	1,364	11.5	1.00 (ref.)	
Yes	3,551	429	10.8	0.82 (0.73–0.92)	DROPPED	387	9.8	0.84 (0.74–0.94)	DROPPED
Missing	23	2	8.0			2	8.0		
Self-reported allergies, sinuses, or hay fever^d^									
No	9,428	1,313	12.2	1.00 (ref.)	1.00 (ref.)	1,164	11.0	1.00 (ref.)	
Yes	4,603	656	12.5	1.02 (0.93–1.13)	DROPPED	587	11.3	1.03 (0.93–1.15)	DROPPED
Missing	23	2	8.0			2	8.0		
Self-reported kidneys, bladder, adrenal gland, or urinary tract problems^d^									
No	11,902	1,704	12.5	1.00 (ref.)	1.00 (ref.)	1,505	11.2	1.00 (ref.)	
Yes	2,129	265	11.1	0.87 (0.76-1.00)	DROPPED	246	10.4	0.91 (0.79–1.05)	DROPPED
Missing	23	2	8.0			2	8.0		
Self-reported lung or other breathing problems^d^									
No	12,188	1,747	12.5	1.00 (ref.)	1.00 (ref.)	1,545	11.3	1.00 (ref.)	
Yes	1,843	222	10.8	0.84 (0.72–0.97)	DROPPED	206	10.1	0.88 (0.75–1.03)	DROPPED
Missing	23	2	8.0			2	8.0		

^a^Cerner and EPQ were used as data sources for this table

^b^Percent of participants who had COVID-19 among all eligible participants, and ^c^percent of participants whose COVID-19 infection was confirmed by diagnostic test among all eligible participants

^d^Pre-existing health conditions that were significantly associated with either the self-reported COVID-19 infection or hospitalization were included in a backward elimination process including the 10 most prevalent health conditions

To further understand the differences in COVID-19 prevalence across racial and ethnic groups, we examined the average marginal effects across the multivariable models and observed that compared to White patients, Asian patients had a 6% lower estimated prevalence of COVID-19 infection, Black/African American patients had a 2% lower estimated prevalence, and patients of other race or multiple races had a similar level of estimated prevalence. Regarding ethnicity, Hispanic patients had a 2% higher estimated prevalence of COVID-19 infection than non-Hispanic patients (Online Resource 4). In the sensitivity analysis using the combined race/ethnicity variable, the odds ratios reported for each category (Non-Hispanic Black/African American, Non-Hispanic Asian, Non-Hispanic Other races and Hispanic, compared to White as the reference group) were of similar magnitude as the odds ratios for Black/African American, Asian, and Other races versus White and Hispanic versus Non-Hispanic ethnicity reported in the final model that included separate race and ethnicity variables (Online Resource 5).

The associations between patient characteristics and COVID-19 related hospitalization are shown in Table 3. Prevalence of COVID-19-related hospitalization increased with age, ranging from 6.6% for those ages 18–25 years to 24.6% for those ages 75 years and older. In contrast to the results observed by sex for COVID-19 history overall, male patients (13.8%) had higher odds of reporting a hospitalization for COVID-19 as compared with female patients (8.4%) (OR_unadjusted_= 1.75, 95% CI = 1.32–2.34). Racial minorities had higher rates of hospitalization as compared to White patients, although these differences were not statistically significant in the unadjusted analysis. Patients who reported a history of cancer or current cancer (OR_unadjusted_=1.44, 95% CI = 1.06–1.99) and other pre-existing health conditions including diabetes/thyroid problems (OR_unadjusted_=1.61, 95% CI = 1.17–2.21), kidney or urinary tract problems (OR_unadjusted_=2.35, 95% CI = 1.65–3.30), and lung or other breathing problems (OR_unadjusted_=2.65, 95% CI = 1.83–3.77) had higher odds of reporting past hospitalization for COVID-19. No significant difference in COVID-19-related-hospitalization was observed by ethnicity or among patients who reported having allergies, sinuses, or hay ever.

**Table 3:** Self-reported history of COVID-19 hospitalization by patient characteristics among new Moffitt Cancer Center patients reporting a history of COVID-19 infection in 2021–2022

Patient Characteristics^a^	Never hospitalized with COVID-19 infection		Ever hospitalized with COVID-19 infection		Unadjusted odds ratios	Multivariable odds ratios
	n	%^b^	n	%^c^	(95% CI)	(95% CI)
Age at survey						
18–25	57	93.4	4	6.6	1.00 (ref.)	1.00 (ref.)
26–35	154	98.1	3	1.9	0.28 (0.05–1.30)	0.28 (0.05–1.31)
36–45	237	92.6	19	7.4	1.14 (0.41–4.05)	1.06 (0.38–3.82)
46–55	370	94.1	23	5.9	0.89 (0.33–3.10)	0.85 (0.31–3.03)
56–64	389	89.2	47	10.8	1.72 (0.67–5.86)	1.61 (0.61–5.58)
65–74	374	87.4	54	12.6	2.06 (0.80–6.98)	1.82 (0.69–6.29)
75 +	181	75.4	59	24.6	4.65 (1.81–15.79)	4.11 (1.54–14.29)
Sex						
Female	1,070	91.6	98	8.4	1.00 (ref.)	1.00 (ref.)
Male	691	86.2	111	13.8	1.75 (1.32–2.34)	1.50 (1.09–2.05)
Missing	1	100.0	0	0.0		
Race						
White	1,468	90.1	161	9.9	1.00 (ref.)	1.00 (ref.)
Asian	27	87.1	4	12.9	1.35 (0.40–3.51)	1.44 (0.33–4.34)
Black/African American	114	86.4	18	13.6	1.44 (0.83–2.37)	2.11 (1.18–3.60)
American Indian or Alaska Native	7	100.0	0	0.0	1.70 (0.96–2.86)	2.61 (1.43–4.54)
Native Hawaiian or Other Pacific Islander	2	100.0	0	0.0		
Other race	70	81.4	16	18.6		
More than 1 race	12	92.3	1	7.7		
Missing	62	87.3	9	12.7		
Ethnicity						
Non-Hispanic	1,463	90.2	159	9.8	1.00 (ref.)	
Hispanic	221	88.4	29	11.6	1.21 (0.78–1.81)	DROPPED
Missing	78	78.8	21	21.2		
Self-reported cancer						
No	640	91.6	59	8.4	1.00 (ref.)	
Yes	1,121	88.3	149	11.7	1.44 (1.06–1.99)	DROPPED
Missing	1	50.0	1	50.0		
Self-reported heart problems or high blood pressure^d^						
No	1,184	91.4	111	8.6	1.00 (ref.)	
Yes	577	85.6	97	14.4	1.79 (1.34–2.40)	DROPPED
Missing	1	50.0	1	50.0		
Self-reported diabetes, thyroid, or gland problems^d^						
No	1,394	90.5	146	9.5	1.00 (ref.)	
Yes	367	85.5	62	14.5	1.61 (1.17–2.21)	DROPPED
Missing	1	50.0	1	50.0		
Self-reported allergies, sinuses, or hay fever^d^						
No	1,170	89.1	143	10.9	1.00 (ref.)	
Yes	591	90.1	65	9.9	0.90 (0.66–1.22)	DROPPED
Missing	1	50.0	1	50.0		
Self-reported kidneys, bladder, adrenal gland, or urinary tract problems^d^						
No	1,547	90.8	157	9.2	1.00 (ref.)	
Yes	214	80.8	51	19.2	2.35 (1.65–3.30)	DROPPED
Missing	1	50.0	1	50.0		
Self-reported lung or other breathing problems^d^						
No	1,586	90.8	161	9.2	1.00 (ref.)	1.00 (ref.)
Yes	175	78.8	47	21.2	2.65 (1.83–3.77)	2.38 (1.61–3.48)
Missing	1	50.0	1	50.0		

^a^Cerner and EPQ were used as data sources for this table

^b^Percent of participants who were not hospitalized due to COVID-19 among those who reported having COVID-19 and ^c^percent of participants who were hospitalized due to COVID-19 among those who reported having COVID-19

^d^Comorbidities were included in the model if they were significantly associated with either the self-reported COVID-19 infection or self-reported hospitalization due to COVID-19 infection in a backward elimination process including the 10 most prevalent comorbidities

Interestingly, the backward elimination procedure implemented on the multivariable model dropped most of the pre-existing health conditions that were significant in the unadjusted models. The final multivariable model revealed that COVID-19 related hospitalization was significantly more prevalent among patients 75 and older (OR = 2.65, 95% CI = 1.83–3.77), males (OR = 1.50, 95% CI = 1.09–2.05), Black/African American patients (OR = 2.11, 95% CI = 1.18–3.60) as well as patients of races other than Black/African American and Asian (OR = 2.61, 95% CI = 1.43–4.54), and patients with lung or other breathing problems (OR = 2.39, 95% CI = 1.61–3.48). The average marginal effects of hospitalization for COVID-19 across racial and ethnic groups were also examined in the multivariable model (Online resource 4). The results suggest that compared to White patients, Asian patients had a 3% higher estimated prevalence of hospitalization, Black/African American patients had an 8% higher estimated prevalence, and patients of other race or multiple races had an 11% higher prevalence. No differences were observed in the average marginal effects of COVID-19 related hospitalization among Hispanic patients (Online Resource 3).

In the sensitivity analysis using the combined race/ethnicity variable, the magnitude of the associations between past COVID-19 hospitalization and non-Hispanic minority race groups (Black/African American, Asian, Other) compared to non-Hispanic Whites were of similar magnitude as the associations observed for the same racial groups in the final model that included separate race and ethnicity variables (Black/African American, Asian, Other compared to White; Hispanic compared to non-Hispanic). However, the association between Hispanic ethnicity and past COVID-19 hospitalization was greater (OR = 2.06) in the sensitivity analysis where Hispanic patients were compared only to non-Hispanic Whites versus in the model where Hispanic patients were compared to non-Hispanic patients of all races, with adjustment for race as a separate variable (Online Resource 6).

## Discussion

Among patients first visiting a comprehensive cancer center between 2021 and 2022, 12.3% reported ever having had COVID-19, which is higher than the 7.8% positivity rate reported in cancer patients receiving care within the US Veterans Affairs (VA) Healthcare system from January-May 2020, and the 1.4% positivity rate reported among French cancer patients from March-May 2020 [27]. However, our findings are lower than Florida’s cumulative population-level positivity rates from this time period which ranged from 16.8% in June 2021 to 26.3% in March of 2022 [28]. Differences in COVID-19 positivity across these studies may be due to differences in population characteristics, local public health measures, COVID-19 strains, and the timing of when infection rates were ascertained. However, as expected, the COVID-19 infection rate increased over time in our study (from 8.6% in the first half of 2021 to 17.5% in early 2022), due in part to greater access to testing and other individual factors [29] while the hospitalization rates decreased (from 10.6 to 3.4% in the same timeframe) as COVID-19 vaccinations and anti-retroviral treatments became more widely available [30, 31].

We observed significant disparities in the prevalence of past COVID-19 infection within our cancer center patient population. Specifically, older adults (56+) had higher odds of reporting history of COVID-19 as compared to adults ages 18–25, inconsistent with a previous study of cancer patients seeking care within the VA system that observed no differences in COVID-19 positivity by age [27]. It is possible that the older patients in the current study were more likely to follow public health guidance on mask wearing and social distancing. Regarding race/ethnicity, Asian patients were less like to report COVID-19 as compared to White patients. Conversely, Hispanic patients had higher odds of reporting history of COVID-19 than non-Hispanic patients, as previously reported in the general population [32–34] and among patients with a cancer history [27]. In our analysis, we included patient reported pre-existing health conditions within the multivariable model examining the association between patient characteristics and COVID-19 infection. However, the pre-existing health conditions were dropped from the final model, suggesting that they do not fully explain the observed demographic differences in COVID-19 positivity rates. Other studies suggest that higher rates of infection in Hispanics may be due to social economic status [34], living conditions not allowing for appropriate social distancing [33, 34], and inability to stay home from work [34, 35].

When examining factors associated with COVID-19 related hospitalization, we observed that that older age and male sex were associated with higher odds of hospitalization, consistent with previous findings in the general population [36] and among patients with cancer [12, 37]. Black/African American patients, Asian patients and patients self-identifying with “other” racial groups had higher odds of being hospitalized with COVID-19 compared to White patients, consistent with other studies of cancer patients [27, 38, 39] and non-cancer patients [18, 40, 41]. Furthermore, the sensitivity analysis using a combined race/ethnicity variable revealed that Hispanic patients were also more likely to experience a COVID-related hospitalization, as observed in previous studies [8, 18, 37]. Lung or other breathing problems was the only category of pre-existing health conditions associated with a higher odds of COVID-19 related hospitalization, corroborating the findings of a meta-analysis in which patients with respiratory disease were four times more likely to have severe COVID-19 outcomes [42]. The cross-sectional design of the questions within the EPQ limited the ability to determine if patients were reporting a history of breathing problems due to COVID-19 infection or if the breathing problems preceded their COVID-19 infection. Nonetheless, the observed racial disparity in past COVID-19 hospitalizations was independent of all pre-existing health conditions in the current study, including lung and breathing problems, and may be due to the complex relationship between race/ethnicity, poverty, and access to care [27, 38, 43]. Future studies are needed to more precisely tease apart factors that may impact risk of COVID-19 related hospitalization among cancer patient populations including demographic characteristics, social economic status, and other clinical factors.

Our study is novel, as self-reported COVID-19 history was collected through an existing institutional electronic data capture system. Data collected through this system along with other institutional databases can be used to understand the prevalence of COVID-19 history among cancer patients, identify potential health disparities, better manage patient care, and examine long-term health outcomes. Our data collection methods may be useful to other researchers or health facilities attempting to rapidly ascertain COVID-19 or other emergent conditions on a large scale. However, while electronic data capture affords the benefit of scale, patient self-reported data may be inaccurate. Since COVID-19 history was ascertained by self-reported data through the EPQ, it is possible that patients misremembered their infection status. Furthermore, COVID-19 history was not available for (~ 21%) patients who did not complete the EPQ, resulting in possible selection bias [44]. Lastly, these data were collected at a single institution and may not be generalizable to patients seeking care at other cancer centers. Future advances in patient-facing digital tools are needed to improve completeness of self-reported data collection in clinical settings across various centers.

Our cross-sectional analysis of patients seeking care at a cancer center ascertained history of COVID-19 infection and hospitalization at the time of first visit to Moffitt. If the factors associated with previous hospitalization for COVID-19 are different than the factors associated with death from COVID-19, then the associations observed in the current study could have been impacted by survival bias and would not be generalizable to the population as a whole. However, the goal of the current study was to assess prevalence of COVID-19 history among patients visiting a cancer center, to provide a baseline for future studies of COVID-19 history and cancer outcomes. This is particularly important since the longer-term impact of COVID-19 on immune function is largely unknown. Some studies suggest that COVID-19 may induce autophagy in cancerous cells which could lead to cancer progression, treatment resistance, or recurrence [23, 24] and cohort studies are underway to examine the impact of COVID-19 on cancer [45, 46]. If previous hospitalization for COVID-19 influences subsequent cancer treatment response and/or outcomes, then the observed associations between demographics and history of COVID-19 hospitalization could portend disparities in cancer outcomes in the future.

In conclusion, patients seeking care at a high-volume academic cancer center who were males, as well as patients who identified as being Black/African American or Hispanic, were most likely to report a history of severe COVID-19 infection, independent of pre-existing conditions. These findings may have implications for subsequent disparities in cancer-related outcomes if past COVID-19 infection is shown to impact longer term cancer outcomes. Additional studies with longer term follow-up will be needed to further investigate COVID-19-associated cancer outcomes and their differences across diverse patient populations.

## Electronic Supplementary Material

Below is the link to the electronic supplementary material.

Supplementary Material 1

## Data Availability

The data underlying this article will be shared upon reasonable request to the corresponding author.
