# Supplementary material for: Factors Associated with Self-reported COVID-19 Infection and Hospitalization among Patients Seeking Care at a Comprehensive Cancer Center
Source: J Racial Ethn Health Disparities. 2023 Nov 2;12(1):107–17. doi: 10.1007/s40615-023-01855-4 (PMC11747054; doi:10.1007/s40615-023-01855-4)
Supplement: Supplementary file 1 — Supplementary Material 1 [file 40615_2023_1855_MOESM1_ESM.docx]

**Title:** Factors associated with self-reported COVID-19 infection and hospitalization among patients seeking care at a comprehensive cancer center

**Journal Name:** Journal of Racial and Ethnic Health Disparities

**Authors:** Rossybelle P. Amorrortu, Research Project Manger^1^, Yayi Zhao, Data Anlayst^1^, Robert J. Keenan, Vice President, Quality and Chief Medical Officer^2^, Scott M. Gilbert, Senior Member^3,4^, Dana Rollison, Senior Member^1^

**Affiliations:**

^1^Department of Cancer Epidemiology, Moffitt Cancer Center, Tampa, FL, 33612, USA

^2^Department of Thoracic Oncology, Moffitt Cancer Center, Tampa, FL, USA

^3^Department of Health Outcomes and Behavior, Moffitt Cancer Center, Tampa, FL, USA

^4^Department of Genitourinary Oncology, Moffitt Cancer Center, Tampa, FL, USA

**Corresponding Author:**

Dana E. Rollison, PhD

Email: Dana.rollison@moffitt.org

Online Resource 1. List of medical problems other than cancer presented to patients when completing the Electronic Patient Questionnaire (EPQ).

| **Medical Conditions** |
| --- |
| Allergies, sinuses, or hay fever |
| Arthritis, autoimmune diseases, or joint problems |
| Bleeding, clotting, or other blood problems |
| Brain and neurological system problems such as stroke, headaches, or seizures |
| Breast or nipple problems |
| Depression, anxiety, or other mental health problems |
| Diabetes, thyroid, or gland problems |
| Digestive tract problems including stomach, colon, bowels, pancreas, liver, and gallbladder |
| Gyn (gynecological) problems including problems with uterus, ovaries, vagina, vulva, and  cervix *(Female Only)* |
| Heart problems or high blood pressure |
| Kidneys, bladder, adrenal gland, or urinary tract problems |
| Lung or other breathing problems |
| Penis, testis, or sperm problems *(Male Only)* |
| Prostate problems *(Male Only)* |
| Skin or mole related problems |
| Other problems |

Online Resource 2. Prevalence of cancer among new patients to Moffitt Cancer Center in 2021-2022 by self-reported history of COVID-19 infection and associated hospitalization.

|  | Number (n) and Prevalence (%) of cancer | | | | | | |
| --- | --- | --- | --- | --- | --- | --- | --- |
|  | by COVID-19 history | | |  | by COVID-19 hospitalization | | |
| Cancer type^a^ | Never had COVID-19 |  | Ever had COVID-19 |  | Never hospitalized with COVID-19^b^ |  | Ever  hospitalized  with  COVID-19^b^ |
|  | n (%) |  | n (%) |  | n (%) |  | n (%) |
| Any cancer | 9740 (69.4) |  | 1270 (64.5) |  | 1121 (63.7) |  | 149 (71.6) |
| Anal | 51 (0.4) |  | 7 (0.4) |  | 7 (0.4) |  | 0 (0) |
| Bladder | 470 (3.3) |  | 66 (3.4) |  | 57 (3.2) |  | 9 (4.3) |
| Brain | 196 (1.4) |  | 26 (1.3) |  | 23 (1.3) |  | 3 (1.4) |
| Breast | 1749 (12.5) |  | 260 (13.2) |  | 237 (13.5) |  | 23 (11.1) |
| Cervical | 159 (2.1) |  | 22 (1.9) |  | 21 (2.0) |  | 1 (1.0) |
| Colon | 462 (3.3) |  | 67 (3.4) |  | 58 (3.3) |  | 9 (4.3) |
| Carcinoid tumor or neuroendocrine tumor | 158 (1.1) |  | 22 (1.1) |  | 19 (1.1) |  | 3 (1.4) |
| Esophageal | 158 (1.1) |  | 23 (1.2) |  | 18 (1.0) |  | 5 (2.4) |
| Gall bladder | 23 (0.2) |  | 1 (0.1) |  | 1 (0.1) |  | 0 (0) |
| Gastrointestinal stromal tumor | 43 (0.3) |  | 5 (0.3) |  | 5 (0.3) |  | 0 (0) |
| Hodgkin's Lymphoma | 144 (1.0) |  | 14 (0.7) |  | 10 (0.6) |  | 4 (1.9) |
| Leukemia | 373 (2.7) |  | 55 (2.8) |  | 45 (2.6) |  | 10 (4.8) |
| Liver | 275 (2.0) |  | 36 (1.8) |  | 28 (1.6) |  | 8 (3.8) |
| Lung | 934 (6.7) |  | 79 (4.0) |  | 66 (3.7) |  | 13 (6.2) |
| Melanoma | 788 (5.6) |  | 94 (4.8) |  | 85 (4.8) |  | 9 (4.3) |
| Multiple myeloma or plasma cell tumor | 288 (2.1) |  | 41 (2.1) |  | 32 (1.8) |  | 9 (4.3) |
| Non-Hodgkin's Lymphoma | 463 (3.3) |  | 47 (2.4) |  | 41 (2.3) |  | 6 (2.9) |
| Oral | 196 (1.4) |  | 20 (1.0) |  | 16 (0.9) |  | 4 (1.9) |
| Ovarian | 187 (2.4) |  | 25 (2.2) |  | 23 (2.2) |  | 2 (2.1) |
| Pancreatic | 325 (2.3) |  | 36 (1.8) |  | 34 (1.9) |  | 2 (1.0) |
| Prostate | 1045 (16.5) |  | 131 (16.2) |  | 119 (17) |  | 12 (10.9) |
| Rectal | 127 (0.9) |  | 8 (0.4) |  | 6 (0.3) |  | 2 (1.0) |
| Kidney (renal cell) | 312 (2.2) |  | 41 (2.1) |  | 33 (1.9) |  | 8 (3.8) |
| Sarcoma | 249 (1.8) |  | 44 (2.2) |  | 40 (2.3) |  | 4 (1.9) |
| Other skin cancer | 1171 (8.3) |  | 127 (6.4) |  | 106 (6) |  | 21 (10.1) |
| Stomach (gastric) | 90 (0.6) |  | 7 (0.4) |  | 4 (0.2) |  | 3 (1.4) |
| Testicular | 72 (1.1) |  | 14 (1.7) |  | 14 (2.0) |  | 0 (0) |
| Thyroid | 319 (2.3) |  | 41 (2.1) |  | 36 (2.0) |  | 5 (2.4) |
| Uterine | 251 (3.3) |  | 37 (3.2) |  | 33 (3.1) |  | 4 (4.2) |
| Other cancer | 1259 (9.0) |  | 154 (7.8) |  | 135 (7.7) |  | 19 (9.1) |
| ^a^EPQ was the data source for this table.  ^b^Among patients who self-reported ever having a COVID-19 infection. | | | | | | | |

Online Resource 3. Prevalence of pre-existing health conditions among new patients to Moffitt Cancer Center in 2021-2022 by self-reported history of COVID-19 infection and associated hospitalization.

| Pre-existing health conditions^a^ | Number (n) and Prevalence (%) of pre-existing health condition | | | | | | |
| --- | --- | --- | --- | --- | --- | --- | --- |
|  | by COVID-19 history | | |  | by COVID-19 hospitalization | | |
|  | Never had  COVID-19 |  | Ever had COVID-19 |  | Never hospitalized with  COVID-19^b^ |  | Ever hospitalized with  COVID-19^b^ |
|  | n (%) |  | n (%) |  | n (%) |  | n (%) |
| Any pre-existing condition | 12019 (85.7) |  | 1604 (81.5) |  | 1427 (81) |  | 177 (85.1) |
| Depression, anxiety, or other mental health problems | 2805 (20.0) |  | 415 (21.1) |  | 372 (21.1) |  | 43 (20.7) |
| Allergies, sinuses, or hay fever | 4603 (32.8) |  | 656 (33.3) |  | 591 (33.6) |  | 65 (31.2) |
| Arthritis, autoimmune diseases, or joint problems | 4233 (30.2) |  | 527 (26.8) |  | 455 (25.8) |  | 72 (34.6) |
| Bleeding, clotting, or other blood problems | 1164 (8.3) |  | 167 (8.5) |  | 134 (7.6) |  | 33 (15.9) |
| Breast or nipple problems | 588 (4.2) |  | 85 (4.3) |  | 80 (4.5) |  | 5 (2.4) |
| Diabetes, thyroid, or gland problems | 3551 (25.3) |  | 429 (21.8) |  | 367 (20.8) |  | 62 (29.8) |
| Gyn (gynecological) problems including problems with uterus, ovaries,  vagina, vulva, and cervix (female only) | 1804 (23.6) |  | 263 (22.8) |  | 241 (22.8) |  | 22 (22.9) |
| Heart problems or high blood pressure | 5893 (42.0) |  | 674 (34.2) |  | 577 (32.8) |  | 97 (46.6) |
| Kidneys, bladder, adrenal gland, or urinary tract problems | 2129 (15.2) |  | 265 (13.5) |  | 214 (12.2) |  | 51 (24.5) |
| Lung or other breathing problems | 1843 (13.1) |  | 222 (11.3) |  | 175 (9.9) |  | 47 (22.6) |
| Brain and neurological system problems such as stroke, headaches, or  seizures | 1271 (9.1) |  | 187 (9.5) |  | 163 (9.3) |  | 24 (11.5) |
| Prostate problems (male only) | 1494 (23.5) |  | 145 (17.9) |  | 131 (18.7) |  | 14 (12.7) |
| Skin or mole related problems | 2433 (17.3) |  | 310 (15.7) |  | 278 (15.8) |  | 32 (15.4) |
| Digestive tract problems including stomach, colon, bowels, pancreas, liver,  and gallbladder | 3203 (22.8) |  | 396 (20.1) |  | 346 (19.6) |  | 50 (24.0) |
| Penis, testis, or sperm problems (male only) | 239 (3.8) |  | 28 (3.5) |  | 23 (3.3) |  | 5 (4.5) |
| Other health problems | 1263 (9.0) |  | 159 (8.1) |  | 145 (8.2) |  | 14 (6.7) |
| ^a^EPQ was the data source for this table.  ^b^Among patients who self-reported ever having a COVID-19 infection. |  |  |  |  |  |  |  |

| Online Resource 4. Average marginal effects from the multivariable logistic regression models examining the association between patient characteristics and self-reported history of COVID-19 infection and COVID-19 associated hospitalization. | | | |
| --- | --- | --- | --- |
| Patient Characteristics | Multivariable logistic regression model for | | |
|  | self-reported COVID-19 history | self-reported COVID-19 history confirmed by diagnostic test | self-reported COVID-19 hospitalization |
| Age (years) |  |  |  |
| 26-35 vs. 18-25 | -0.01 | -0.01 | -0.05 |
| 36-45 vs. 18-25 | -0.04 | -0.04 | 0.00 |
| 46-55 vs. 18-25 | -0.05 | -0.04 | -0.01 |
| 56-64 vs. 18-25 | -0.09 | -0.09 | 0.04 |
| 65-74 vs. 18-25 | -0.14 | -0.12 | 0.05 |
| 75+ vs. 18-25 | -0.15 | -0.14 | 0.16 |
| Gender |  |  |  |
| Male vs. Female |  |  | 0.04 |
| Race |  |  |  |
| Asian vs. White | -0.06 | -0.05 | 0.03 |
| Black vs. White | -0.02 | -0.01 | 0.08 |
| Other/multiple vs. White | 0.00 | 0.01 | 0.11 |
| Ethnicity |  |  |  |
| Hispanic/Latino vs. Non-Hispanic | 0.02 | 0.03 |  |
| Self-reported lung or other breathing problems |  |  |  |
| Yes vs. No |  |  | 0.08 |

Online Resource 5. Multi-variable model of self-reported history of COVID-19 infection by patient characteristics with combined race and ethnicity variable among new Moffitt Cancer Center patients in 2021- 2022.

| Patient Characteristics^a^ | Self-reported COVID-19 Infection | | | | | | | | | |
| --- | --- | --- | --- | --- | --- | --- | --- | --- | --- | --- |
|  | Never had COVID-19 |  | Ever had COVID-19 | | Multivariable odds ratios |  | COVID-19 confirmed by diagnostic test | | Multivariable odds ratios |  |
|  | n |  | n | %^b^ | (95% CI) |  | n | %^c^ | (95% CI) |  |
| Age at survey |  |  |  |  |  |  |  |  |  |  |
| 18 - 25 | 213 |  | 61 | 22.3 | 1.00 (ref.) |  | 55 | 20.5 | 1.00 (ref.) |  |
| 26 - 35 | 570 |  | 157 | 21.6 | 0.92 (0.66-1.31) |  | 141 | 19.8 | 0.93 (0.65-1.34) |  |
| 36 - 45 | 1,160 |  | 256 | 18.1 | 0.76 (0.55-1.05) |  | 225 | 16.2 | 0.75 (0.54-1.06) |  |
| 46 - 55 | 1,822 |  | 393 | 17.7 | 0.72 (0.53-1.00) |  | 352 | 16.2 | 0.73 (0.53-1.02) |  |
| 56 - 64 | 2,908 |  | 436 | 13.0 | 0.52 (0.38-0.71) |  | 377 | 11.5 | 0.51 (0.37-0.71) |  |
| 65 - 74 | 4,274 |  | 428 | 9.1 | 0.33 (0.25-0.46) |  | 387 | 8.3 | 0.34 (0.25-0.48) |  |
| 75 + | 3,107 |  | 240 | 7.2 | 0.26 (0.19-0.36) |  | 216 | 6.5 | 0.27 (0.19-0.38) |  |
| Sex |  |  |  |  |  |  |  |  |  |  |
| Female | 7,728 |  | 1,168 | 13.1 |  |  | 1,047 | 11.9 |  |  |
| Male | 6,322 |  | 802 | 11.3 | DROPPED |  | 705 | 10.0 | DROPPED |  |
| Missing | 4 |  | 1 | 20.0 |  |  | 1 | 20.0 |  |  |
| Race and ethnicity |  |  |  |  |  |  |  |  |  |  |
| Non-Hispanic White | 10,364 |  | 1,426 | 12.1 | 1.00 (ref.) |  | 1,249 | 10.8 | 1.00 (ref.) |  |
| Non-Hispanic Asian | 342 |  | 30 | 8.1 | 0.50 (0.33-0.72) |  | 30 | 8.1 | 0.57 (0.38-0.82) |  |
| Non-Hispanic Black/African American | 798 |  | 118 | 12.9 | 0.84 (0.68-1.03) |  | 108 | 11.9 | 0.88 (0.71-1.09) |  |
| Non-Hispanic other race/multiple races | 199 |  | 35 | 15.0 | 0.99 (0.67-1.41) |  | 33 | 14.2 | 1.07 (0.72-1.54) |  |
| Hispanic | 1,153 |  | 250 | 17.8 | 1.23 (1.05-1.43) |  | 236 | 17.0 | 1.33 (1.14-1.55) |  |
| Missing | 1,198 |  | 112 | 8.5 |  |  | 97 | 7.5 |  |  |
| Self-reported lung or other breathing problems^d^ |  |  |  |  |  |  |  |  |  |  |
| No | 12,188 |  | 1,747 | 12.5 |  |  | 1,545 | 11.3 |  |  |
| Yes | 1,843 |  | 222 | 10.8 | DROPPED |  | 206 | 10.1 | DROPPED |  |
| Missing | 23 |  | 2 | 8.0 |  |  | 2 | 8.0 |  |  |

^a^Cerner and EPQ were used as data sources of this table.

^b^Percent of participants who had COVID-19 among all eligible participants, and ^c^percent of participants whose COVID-19 infection was confirmed by diagnostic test among all eligible participants.

^d^Pre-existing health conditions that were significantly associated with either the self-reported COVID-19 infection or hospitalization were included in a backward elimination process including 10 most prevalent health conditions.

Online Resource 6. Multi-variable model of history of COVID-19 hospitalization by patient characteristics with combined race and ethnicity variable among new Moffitt Cancer Center patients reporting a history of COVID-19 infection in 2021- 2022.

| Patient Characteristics^a^ | Never hospitalized with COVID-19 infection | | Ever hospitalized with COVID-19 infection | | Multivariable odds ratios |  |
| --- | --- | --- | --- | --- | --- | --- |
|  |  |  |  |  |  |  |
|  | n | %^b^ | n | %^c^ | (95% CI) |  |
| Age at survey |  |  |  |  |  |  |
| 18 - 25 | 57 | 93.4 | 4 | 6.6 | 1.00 (ref.) |  |
| 26 - 35 | 154 | 98.1 | 3 | 1.9 | 0.31 (0.06- 1.48) |  |
| 36 - 45 | 237 | 92.6 | 19 | 7.4 | 1.09 (0.38- 3.94) |  |
| 46 - 55 | 370 | 94.1 | 23 | 5.9 | 0.85 (0.30- 3.03) |  |
| 56 - 64 | 389 | 89.2 | 47 | 10.8 | 1.77 (0.67- 6.13) |  |
| 65 - 74 | 374 | 87.4 | 54 | 12.6 | 1.92 (0.72- 6.68) |  |
| 75 + | 181 | 75.4 | 59 | 24.6 | 4.58 (1.70-16.08) |  |
| Sex |  |  |  |  |  |  |
| Female | 1,070 | 91.6 | 98 | 8.4 | 1.00 (ref.) |  |
| Male | 691 | 86.2 | 111 | 13.8 | 1.56 (1.12- 2.16) |  |
| Missing | 1 | 100.0 | 0 | 0.0 |  |  |
| Race and ethnicity |  |  |  |  |  |  |
| Non-Hispanic White | 1,293 | 90.7 | 133 | 9.3 | 1.00 (ref.) |  |
| Non-Hispanic Asian | 26 | 86.7 | 4 | 13.3 | 1.69 (0.39- 5.17) |  |
| Non-Hispanic Black/African American | 101 | 85.6 | 17 | 14.4 | 2.61 (1.42- 4.60) |  |
| Non-Hispanic other race/multiple races | 30 | 85.7 | 5 | 14.3 | 2.39 (0.78- 6.06) |  |
| Hispanic | 201 | 87.4 | 29 | 12.6 | 2.06 (1.29- 3.23) |  |
| Missing | 91 | 81.3 | 21 | 18.8 |  |  |
| Self-reported lung or other breathing problems^d^ |  |  |  |  |  |  |
| No | 1,586 | 90.8 | 161 | 9.2 | 1.00 (ref.) |  |
| Yes | 175 | 78.8 | 47 | 21.2 | 2.41 (1.61- 3.57) |  |
| Missing | 1 | 50.0 | 1 | 50.0 |  |  |

^a^Cerner and EPQ were used as data sources of this table.

^b^Percent of participants who had COVID-19 among all eligible participants, and ^c^percent of participants whose COVID-19 infection was confirmed by diagnostic test among all eligible participants.

^d^Pre-existing health conditions that were significantly associated with either the self-reported COVID-19 infection or hospitalization were included in a backward elimination process including 10 most prevalent health conditions.
